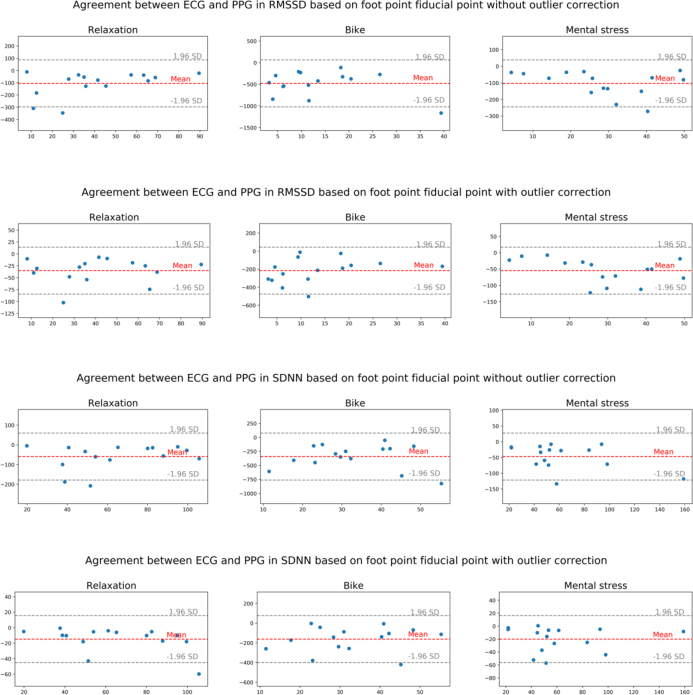# Estimation of Heart Rate Variability from Finger Photoplethysmography During Rest, Mild Exercise and Mild Mental Stress

**DOI:** 10.2478/joeb-2021-0012

**Published:** 2021-12-18

**Authors:** Bjørn-Jostein Singstad, Naomi Azulay, Andreas Bjurstedt, Simen S. Bjørndal, Magnus F. Drageseth, Peter Engeset, Kari Eriksen, Muluberhan Y. Gidey, Espen O. Granum, Matias G. Greaker, Amund Grorud, Sebastian O. Hewes, Jie Hou, Adrián M. Llop Recha, Christoffer Matre, Arnoldas Seputis, Simen E. Sørensen, Vegard Thøgersen, Vegard Munkeby Joten, Christian Tronstad, Ørjan G. Martinsen

**Affiliations:** 1 Department of Physics, University of Oslo, Oslo, Norway; 2 Department of Research and Development, Division of Emergencies and Critical Care, Oslo University Hospital, Oslo, Norway; 3 Institute of Clinical Medicine, University of Oslo, Oslo, Norway; 4 Department of Clinical and Biomedical Engineering, Oslo University Hospital, Oslo, Norway

**Keywords:** electrocardiography, plethysmography, heart rate variability

## Abstract

Due to the possibilities in miniaturization and wearability, photoplethysmography (PPG) has recently gained a large interest not only for heart rate measurement, but also for estimating heart rate variability, which is derived from ECG by convention. The agreement between PPG and ECG-based HRV has been assessed in several studies, but the feasibility of PPG-based HRV estimation is still largely unknown for many conditions. In this study, we assess the feasibility of HRV estimation based on finger PPG during rest, mild physical exercise and mild mental stress. In addition, we compare different variants of signal processing methods including selection of fiducial point and outlier correction. Based on five minutes synchronous recordings of PPG and ECG from 15 healthy participants during each of these three conditions, the PPG-based HRV estimation was assessed for the SDNN and RMSSD parameters, calculated based on two different fiducial points (foot point and maximum slope), with and without outlier correction. The results show that HRV estimation based on finger PPG is feasible during rest and mild mental stress, but can give large errors during mild physical exercise. A good estimation is very dependent on outlier correction and fiducial point selection, and SDNN seems to be a more robust parameter compared to RMSSD for PPG-based HRV estimation.

## Introduction

PPG is a method that measures the change in volume by using light. This method can be used to measure the pulsating dilatation of blood vessels, caused by the heart, by attaching a PPG sensor to the skin. The PPG sensor transmits a light signal which is reflected by or transmitted through the blood vessels. The reflected or transmitted light from the area covered by the PPG light source provides a pulsating signal, which is picked up by a photodiode. The pulsating component of the PPG signal is synchronous to the heartbeat but has a delay corresponding to the transit time of the blood from the heart to the point of measurement [1,2]. In this way, the PPG signal can be used to derive the heart rate.

Heart rate variability (HRV) is related to the interaction between the sympathetic and the parasympathetic nervous system [3]. ECG has for a long time been used as the preferred method for measuring HRV, but in recent years, PPG has also been increasingly considered as a method to estimate HRV. Perhaps one of the strongest motivations for using PPG is to reach out to the consumer market as the PPG sensors are simple, low-cost and a comfortable technique [4,5]. The technique for measuring heart rate from PPG is already widely used in Fitbit devices, smartwatches and smartphones [6].

Several studies have demonstrated good performance in estimation of heart rate based on PPG, even during intensive exercise [7], but the potential applicability of PPG for HRV estimation under different conditions is not as clear. Estimation of heart rate by wearable sensors is generally an easier task than estimation of HRV. The temporal changes of interest for heart rate is rarely over less than minutes, allowing ample time for signal processing and filtering, while several methods of HRV calculation uses the variation between consecutive beats. Several studies have compared HRV derived from PPG with ECG-based HRV as a reference [8, 9, 10] and good agreement has generally been demonstrated for healthy subjects at rest, but moderate physical, and sometimes mental stress, tends to diminish the agreement [8,11,12]. A study conducted in 2015 found the correlation between ECG and finger-PPG derived HRV-features to decrease when the subjects were doing exercise on a stationary bike compared with the same subjects at rest [11]. Another study found the correlation between ECG and finger-PPG derived HRV-features to diminish at mental stress compared to the same subjects at rest [13]. To our extent of knowledge only one study has compared the feasibility of finger-PPG-based HRV estimation during rest, exercise and mental stress in the same study. They compared the correlation between ECG and PPG derived frequency-domain features and found no drastic changes in correlation between mental stress and rest, but exercise tended to decrease the correlation between ECG and PPG derived HRV [12]. However, the difference in correlation between ECG and PPG based HRV estimation caused by mental stress and exercise has not been thoroughly elucidated and needs further research [14].

The signal processing of the PPG signal plays an important role in comparison to ECG-based HRV. In this study, we assess the feasibility of PPG-based HRV estimation during rest, mild exercise and mild mental stress. Our approach was to record finger PPG and ECG simultaneously from healthy volunteers during each of these conditions, calculate the most common time-domain HRV parameters based on both ECG and PPG, and assess the performance of PPG-derived HRV parameters based on statistical analysis. In addition, we evaluate different variants of signal processing for deriving the inter-beat interval time series used for calculating the HRV parameters, including the selection of fiducial point and outlier correction.

This study aimed to assess the agreement between heart rate variability derived from finger PPG and ECG during rest, mild exercise and mild mental stress and comparing different signal processing methods.

## Materials and methods

### Equipment

Two Shimmer3 sensor platforms (Shimmer, Dublin, Ireland), with built-in data loggers, were used to collect data from a PPG sensor and taking ECG measurements simultaneously.

The Consensys Pro software (v1.2.0, Shimmer, Dublin, Ireland) was used to extract the data and export it to a readable format. Consensys Base 6 was connected to transfer the data from the wireless Shimmer3 sensor platform to the Consensys Pro software.

The PPG sensor used in this study contains a super bright green light-emitting diode (LED) and an ambient light sensor that detects the reflected light from the blood vessels. The sensor was connected to the Shimmer3 GSR+ sensor platform with a 3.5mm jack cable. The voltage created by the light sensor was read and converted by the Shimmer ADC to a 12-bit number that represents the PPG-signal in mV.

**Figure 1:**
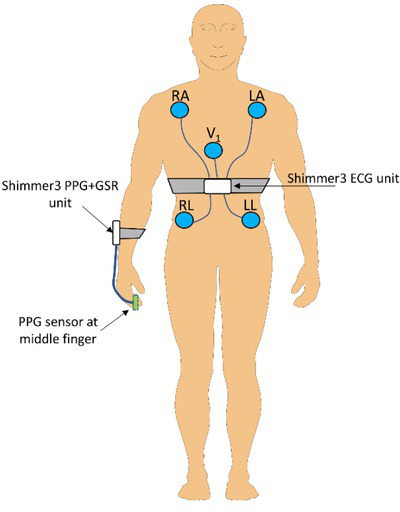
Placement of Shimmer3 measurement units (white boxes), ECG electrodes (blue circles) and the PPG sensor (green box).

The electrodes used for ECG measurement was the Q00A wet gel electrode from Ambu (Ballerup, Denmark). The Shimmer3 ECG unit has an ECG front end which contains an electromagnetic interference (EMI) filter that reduces EMI, and a configurable gain. We used the default gain setting for these measurements (level 6). The Shimmer3 ECG unit also have a respiration demodulation function and a lead-off detection, but this was not used during this study. Both the PPG and ECG signals were sampled at 1kHz. The ECG signal from the LL (left leg)-RA (right arm) lead was used for the HRV analysis. The placement of the sensors and ECG electrodes is shown in Figure 1.

### Subjects and protocol

Twenty-one healthy subjects participated in this study. A majority of them were male (18) and 3 of them were female. The average age of the participants was 26.3 years (SD = 6.2 years), the average height was 180.8 cm (SD = 9.6 cm) and the average weight was 74 kg (SD = 12.4 kg). All participants were informed and gave written consent before the test was initiated. During the test, the participants wore five electrodes to be used for ECG recording. The skin was disinfected with an alcohol swab before the electrodes were attached to ensure good conductivity. The electrodes were placed according to the five-lead Mason-Likar system with the V-lead at V1 [15]. The ECG unit (Shimmer3) was attached to the participant with a chest strap, and each electrode was connected to the ECG unit with wires. The PPG sensor was positioned with a strap around the fingertip of the middle finger on one of the participants hands (optional side). The sensor was connected, with wire, to the GSR+ Shimmer3, attached with a strap to the lower arm of the participant.

The participants underwent three phases during the measurement. In the first phase, the participant sat relaxed in a chair and rested for five minutes during measurement. In phase two, the participant was asked to cycle with low intensity on an ergometer bike for five minutes during the measurement. In the final phase, the participant was asked to play a game designed to measure the signals during mental stress. To give the test subjects a controlled level of mild mental stress, the python-based program LARA was used [16]. This is a simple game where the players are told to look for a specific number in a 6 by 6 matrix and determine how many times the numbers occur in the matrix. Each round is timed, and the player’s score is improved by quicker responding rates. The goal of the game is to complete as many matrixes as possible within three minutes.

### Signal processing

**Figure 2:**
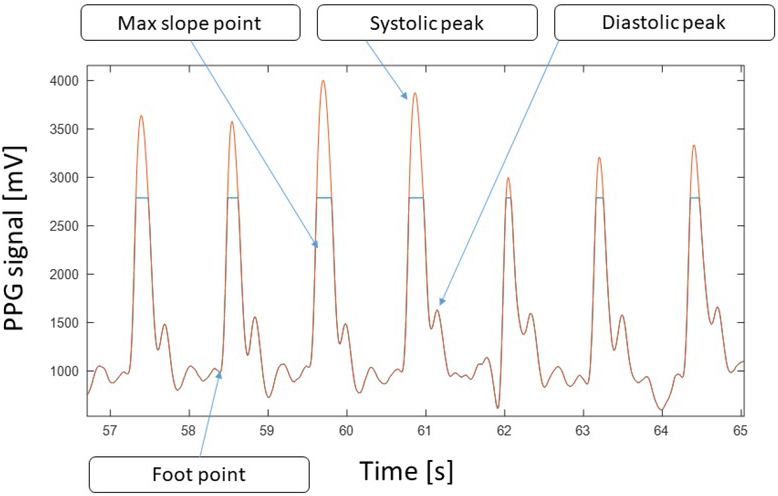
A seven beat PPG sequence showing clipping of the systolic peaks (blue curve) and the spline-based interpolation used (orange curve). The two fiducial points assessed is also marked in the figure.

Initial inspection of the recordings revealed that saturation of the PPG signal would often occur, clipping the signal at around 2800 mV amplitude as shown in Figure 2 (blue lines). This distortion hampered the possibility to assess the PPG-based HRV when using the signal peak point as the fiducial point in calculating the inter-beat intervals. However, a rough estimate of the peak amplitude is useful in the separation between systolic and diastolic peaks, as their amplitude difference may diminish when the systolic peak is clipped. In order to roughly estimate the peak amplitude, any clipped signal parts were first identified by signal values exceeding 2700 mV. These signal parts were then reconstructed by cubic spline interpolation (using the spline() function in Matlab) between the clipped endpoints as shown in Figure 2.

A Savitzky-Golay filtered (5th order, frame length of 51) version of the signal was then differentiated in order to obtain the first derivative of the PPG signal (dPPG). The dPPG was then used to locate negative to positive zero-crossing points of the first derivative, representing PPG signal valleys. These locations were then filtered by excluding those having a too small subsequent PPG wave (the following dPPG peak less than 0.5 mV/ms). Further exclusion of false points was done by adaptive threshold-based filtering (subsequent dPPG peaks had to be larger than a third of the 95% percentile of dPPG peaks within the preceding 10s window, and the PPG valley to peak amplitude had to be larger than 75% of the previous one). Comparison of the current PPG peak to the previous and next peak was then done, excluding points where the PPG peak is lower than 75% of both the previous and next PPG peak, as these are more likely diastolic peaks.

**Figure 3:**
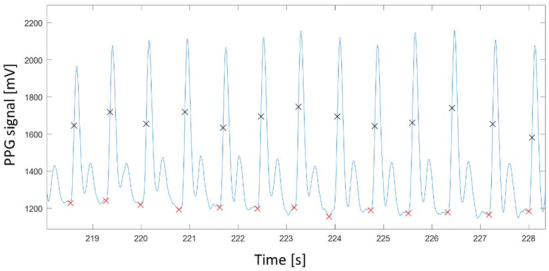
Example showing 13 beats of PPG recording together with the detected foot (red crosses) and max slope (black crosses) fiducial points. The foot points were located based on identifying negative-to-positive zero-crossing points of the first derivative of the signal, and the subsequent peak of the derivative was used to locate the max slope fiducial point.

The filtered vector of PPG valleys was then used as locations of the PPG foot fiducial point, and the locations of the subsequent dPPG peak was used as the PPG max slope fiducial point (figure 3). The vectors of these fiducial points were used to derive inter-beat interval time-series for calculation of HRV parameters.

The ECG-based RR intervals were located by employing the Pan-Tompkins algorithm [17] using a publicly available Matlab® script [18]. Finally, the time differences between neighboring fiducial points of the PPG and ECG time series were used to calculate the IBI and RR vectors respectively.

The time-stamp of the recordings had occurrences of time steps far larger than that of the sampling rate along with signal distortion, and beats belonging to these were identified by detection of time step above 100 ms within a beat. For both the RR and IBI time-series, these beats were replaced by a linear interpolation between the points before and after.

Missing detection or erroneous identification of fiducial points will often lead to outliers in the IBI time vector, which can possibly be identified and filtered automatically. In order to assess the importance of such filtering, an outlier correction filter similar to [19] was implemented as follows: Using a moving window from ten points before to ten points after the current beat, the median and inter-quartile range was calculated. The IBI at the current beat was detected as an outlier if the IBI was more than three interquartiles away from the median IBI, and replaced by linear interpolation between the points before and after.

### Statistical analysis

Based on the IBI and RR vectors, the widely used HRV parameters SDNN (standard deviation among all beats within the interval, representing slow variability) and RMSSD (root mean square of successive differences, representing beat-to-beat variability) were calculated. These parameters were calculated for each participant and every phase, based on the RR vectors and both the unfiltered and filtered IBI vectors. Agreement with ECG-based HRV in each phase was assessed by calculating the median absolute deviation (MAD), the median absolute relative deviation (MARD) and the root mean square error (RMSE) between PPG-based and ECG-based HRV over all subjects. Bland-Altman plots were also constructed for each comparison of HRV calculation. In addition, and for relevance to heart rate measurement, a comparison between the PPG-derived IBI and the ECG-derived RR intervals was done based on the averages within five-second intervals of each recording, presented as MAD, MARD and RMSE. These error measures were first calculated over all five-second intervals within each recording, and the median was then taken over all subjects.

### Informed consent

Informed consent has been obtained from all individuals included in this study.

### Ethical approval

The research related to human use has been complied with all relevant national regulations, institutional policies and in accordance with the tenets of the Helsinki Declaration.

## Results

**Figure 4:**
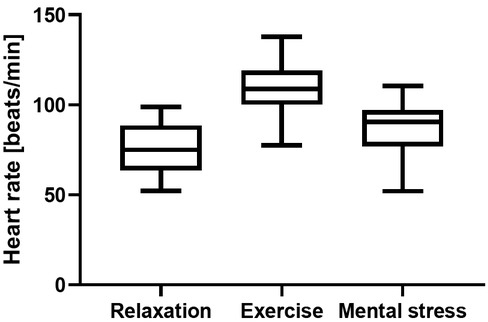
Average heart rate for all participants during the different periods.

The average heart rate within each period (figure 4), shows that the exercise caused a moderate increase in the heart rate (from 75.0 to 109.0 median beats/min), and that the mental stress period also caused an elevation in heart rate (at 90.5 median beats/min), indicating mild exercise for the second period and that a mild level of stress was induced during the third period.

**Figure 5:**
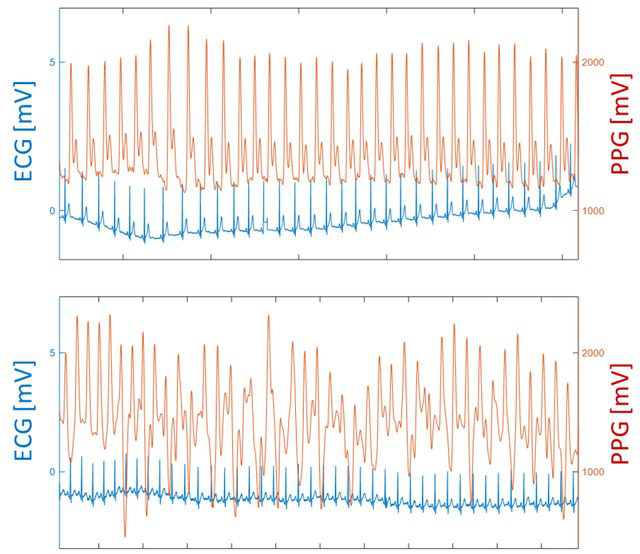
Example segments from recordings with good PPG signal quality (a), during the resting phase, and with poor PPG signal quality (b) during the exercise phase.

**Figure 6:**
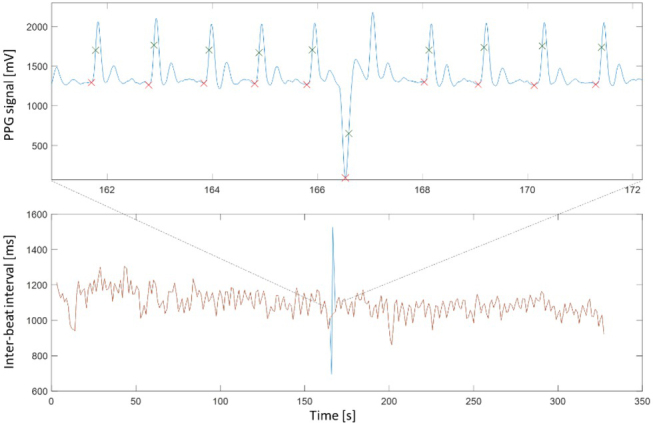
A transient disturbance in the PPG signal (upper plot) causing a large deviation in the estimated inter-beat interval over two points (blue line in lower plot). The red line shows the IBI time-series after outlier correction. The red and green crosses in the upper plot mark the foot and maximum slope fiducial points, respectively.

Initial inspections revealed that one session had missing ECG data and five sessions had a malfunctioning acquisition of PPG signals in all three phases, and had to be excluded from the analysis, resulting in 15 sessions with three complete phases used in the analysis. Visual inspection indicated that the signal quality of PPG was generally good in the resting and mental stress phases, but poor in the exercise phase (see examples in figure 5). Even in sessions with generally good signal quality, transient signal disturbances could cause large deviations in the inter-beat intervals. An example of this is shown in figure 6, where a PPG signal disturbance causes two outliers, which were detected by the filter and replaced by linear interpolation. In this example, the SDNN was 66.8 ms with outlier correction and 74.8 ms without, while the ECG-based SDNN was 65.2.

**Figure 7:**
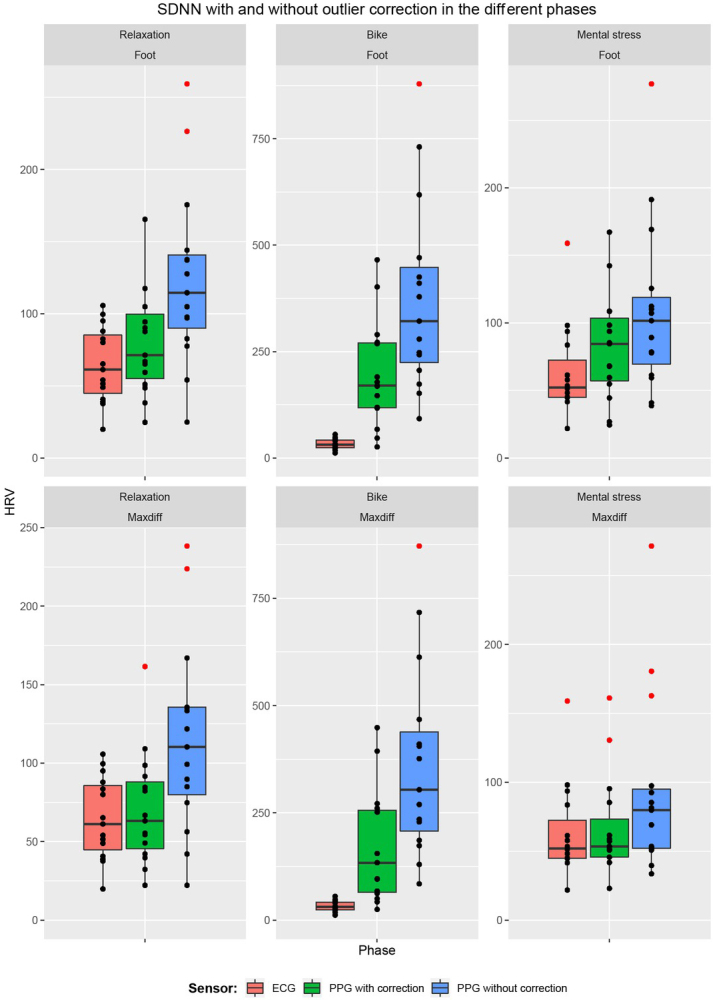
The spread of SDNN for the 15 participants measured with ECG and PPG with and without outlier correction, broken down by the different phases and fiducial points (foot and maxdiff). Outliers are shown in red.

**Figure 8:**
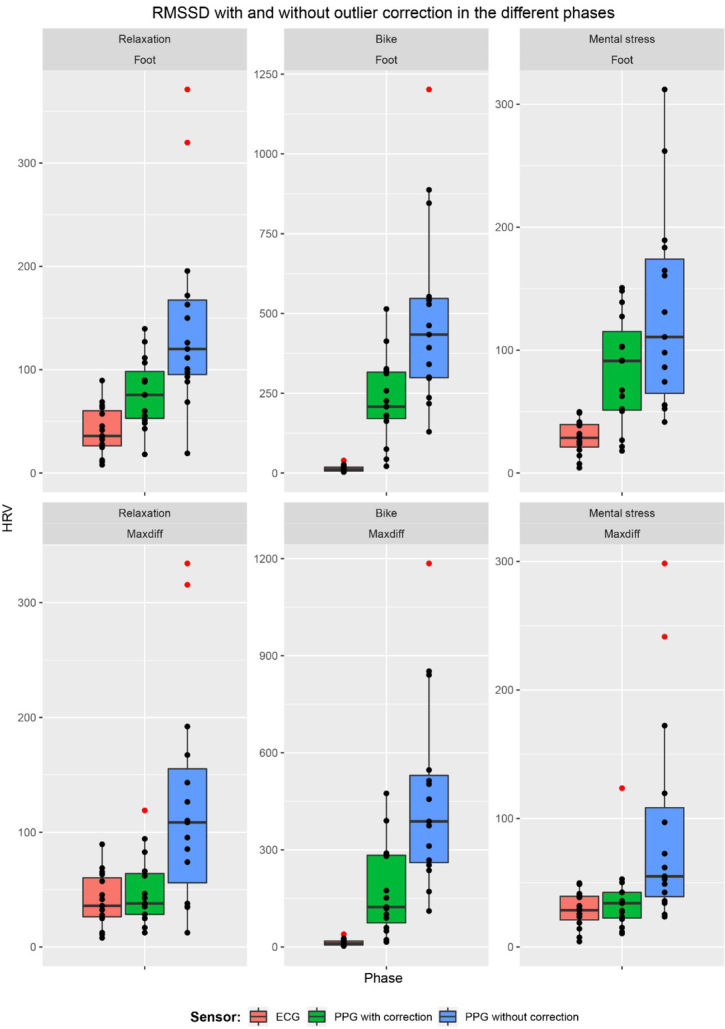
The spread of RMSSD for the 15 participants measured with ECG and PPG with and without outlier correction, broken down by the different phases and fiducial points (foot and maxdiff). Outliers are shown in red.

Figures 7 and 8 provide an overview of the PPG-based SDNN and RMSSD estimates, respectively. It can be seen that the PPG-based approach overestimated the HRV in all cases, from a small overestimation during the rest and stress phases (when using the max slope fiducial point with outlier correction), to a huge overestimation during the exercise period.

**Figure 9:**
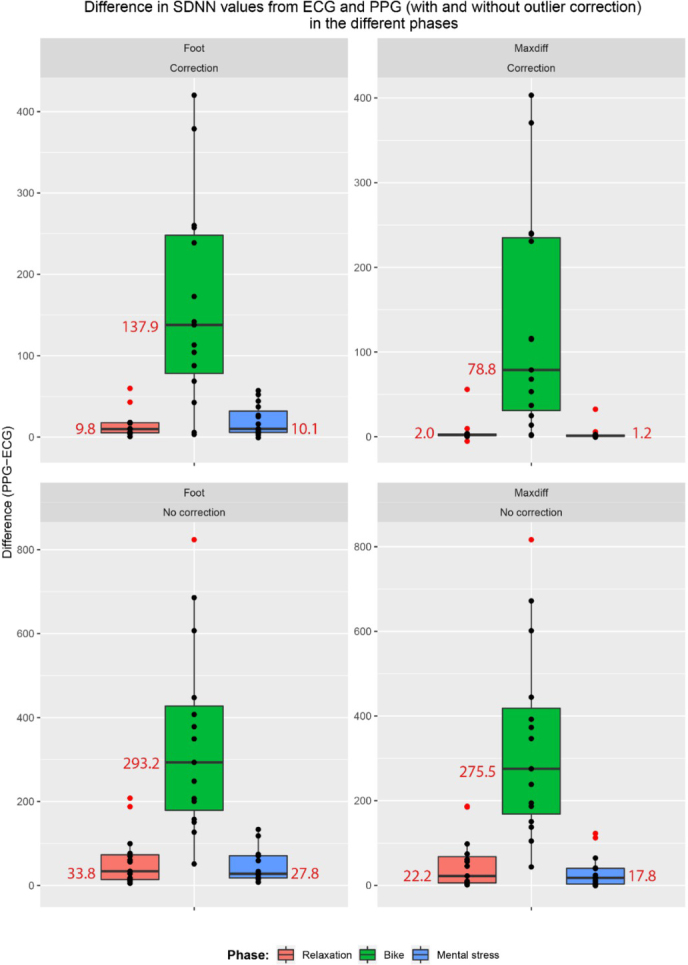
The difference between SDNN values as calculated from PPG and ECG for the 15 participants in the different phases. Grouped by fiducial point and outlier correction. Outliers and median values are shown in red.

**Figure 10:**
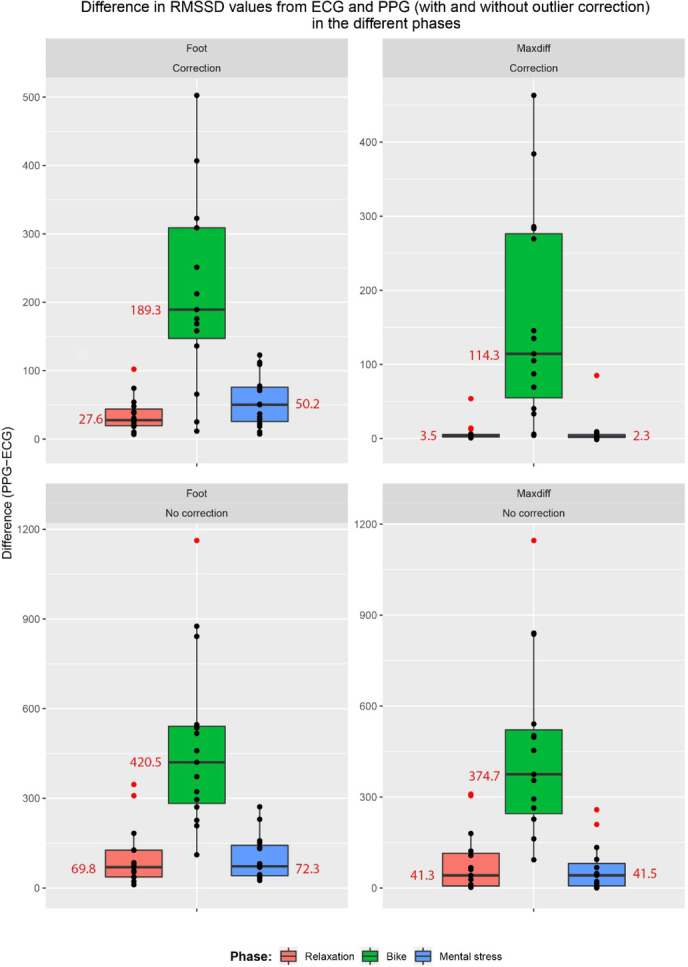
The difference between RMSSD values as calculated from PPG and ECG for the 15 participants in the different phases. Grouped by fiducial point and outlier correction. Outliers and median values are shown in red.

Table 1 gives a summary of the difference between PPG and ECG-derived HRV, depending on the type of activity, selection of fiducial point and correction of outliers. Figures 9 and 10 gives a visual representation of this difference for SDNN and RMSSD respectively. Comparing the MAD and MARD values, the exercise phase clearly had a much larger error than the resting and mental stress phases. There was also a large influence of the outlier correction, with large errors in PPG-based HRV estimation when outliers were not corrected. The average proportions of outlier beats were 1.09% (resting), 4.24% (exercise) and 1.57% (mental stress) when the foot fiducial point was used, and 0.60% (resting), 5.30% (exercise) and 0.73% (mental stress) when the maximum slope fiducial point was used.

**Table 1:** Summary of agreement between HRV derived from ECG and PPG for each type of activity, depending on PPG fiducial point selection with and without correction of outliers. The agreement is represented as median absolute deviation (MAD), median absolute relative deviation (MARD) and the root mean square error (RMSE).

Fiducial point	Outlier correction	HRV	Agreement	Relaxation	Exercise	Mental stress
Foot	Yes	SDNN	MAD	9,83	137,86	10,14
Max slope	Yes	SDNN	MAD	2,25	78,82	1,19
Foot	No	SDNN	MAD	28,12	290,49	26,95
Max slope	No	SDNN	MAD	21,98	272,73	17,6
Foot	Yes	RMSSD	MAD	27,56	189,3	50,17
Max slope	Yes	RMSSD	MAD	3,46	114,26	2,27
Foot	No	RMSSD	MAD	64,21	415,58	72,18
Max slope	No	RMSSD	MAD	41,31	369,77	27,77
Foot	Yes	SDNN	MARD	18,00 %	341 %	23,20 %
Max slope	Yes	SDNN	MARD	3,60 %	239 %	1,80 %
Foot	No	SDNN	MARD	51,50 %	933 %	53,90 %
Max slope	No	SDNN	MARD	22,00 %	877 %	28,30 %
Foot	Yes	RMSSD	MARD	85,00 %	1576 %	156 %
Max slope	Yes	RMSSD	MARD	8,20 %	647 %	6,40 %
Foot	No	RMSSD	MARD	152 %	1727 %	216 %
Max slope	No	RMSSD	MARD	60,00 %	1572 %	79,30 %
Foot	Yes	SDNN	RMSE	21,41	202,95	27,33
Max slope	Yes	SDNN	RMSE	14,83	184,50	8,59
Foot	No	SDNN	RMSE	85,16	403,71	60,56
Max slope	No	SDNN	RMSE	79,17	395,91	49,43
Foot	Yes	RMSSD	RMSE	43,17	253,67	66,01
Max slope	Yes	RMSSD	RMSE	14,98	212,15	22,28
Foot	No	RMSSD	RMSE	143,70	552,46	126,00
Max slope	No	RMSSD	RMSE	131,14	533,94	99,57

Comparing the fiducial points, the Max slope method gave consistently a better agreement than the Foot point. Based on the relative deviation (MARD), the PPG-based estimates of SDNN were more correct than the RMSSD estimates. Taking all cases into account, the best agreement for both SDNN and RMSSD was for Max slope based HRV estimation with outlier correction during the resting and mental stress phases.

The agreement between the R-to-R (RR) intervals derived from ECG and the inter-beat intervals (IBI) derived from the PPG is provided in table 2. This also shows a good agreement for the resting and mental stress tasks and a worse agreement during exercise, and also a reduction in error with the maximum slope fiducial point and from outlier correction.

**Table 2:** Summary of agreement between RR derived from ECG and IBI derived from PPG for each type of activity. The agreement is presented as median absolute deviation (MAD), median absolute relative deviation (MARD) and the root mean square error (RMSE).

Fiducial point	Outlier correction	Agreement	Relaxation	Exercise	Mental stress
Foot	Yes	MAD	3.97	18.93	4.71
Max slope	Yes	MAD	2.82	4.34	2.29
Foot	No	MAD	4.03	42.40	4.46
Max slope	No	MAD	2.93	48.86	2.29
Foot	Yes	MARD	0.51%	3.24%	0.69%
Max slope	Yes	MARD	0.36%	0.67%	0.35%
Foot	No	MARD	0.58%	8.02%	0.62%
Max slope	No	MARD	0.38%	9.45%	0.35%
Foot	Yes	RMSE	13.02	106.27	12.73
Max slope	Yes	RMSE	8.41	99.51	6.98
Foot	No	RMSE	34.03	255.14	21.85
Max slope	No	RMSE	35.15	262.89	14.22

## Discussion

Our results show that HRV estimation based on PPG is strongly dependent on the type of activity and the method of signal processing. The maximum slope as a fiducial point performed better than the foot point of the PPG signal.

Outliers due to artifacts gave rise to large errors if uncorrected before HRV calculation. When using the maximum slope as fiducial point together with outlier correction, good agreement was found for both SDNN and RMSSD during the resting and mental stress periods.

As shown in Table 1, our HRV calculation based on the maximum slope gave a more accurate estimate than the foot point, especially for the RMSSD parameter. This is due to variation of the PPG signal floor which can cause a less defined foot point compared to the unambiguous maximum slope of the rising pulse wave in the systolic phase. Examples of this can be seen from figure 3 and 6, where the foot point location sometimes shifts to the left (around 224s in figure 3 and 170s in figure 6) due to waveform variation from beat to beat.

The large overestimation of HRV during exercise was due to movement artifacts hampering the signal quality by including waves that are difficult to discriminate from the true waves of pulsation. It has been shown that the combined use of an accelerometer signal together with PPG can improve heart rate estimation [20], but this is more difficult when estimating HRV when the changes from beat to beat are of importance. When a continuous disturbance (e.g. running) is added to the PPG signal, large portions of the time-series may be corrupted, and methods such as interpolation may greatly reduce the variability of the inter-beat intervals. The accelerometer signal can however be useful in assessing the quality of PPG signals when used for HRV estimation and avoid HRV analysis based on unreliable data [21,22].

As demonstrated in Figure 6, short transient disturbances in the PPG signal may cause large erroneous deviations in the inter-beat intervals and consequently errors in the HRV estimation. Most of these deviations were easy to detect as outliers based on a simple distance-to-median rule, but become problematic when occurring frequently as during exercise. The outlier correction method we employed was not able to provide a useful estimation of HRV during exercise.

We used a finger placement of the PPG sensor rather than the wrist even though wrist-worn devices are most used by commercial wearable manufacturers. However, some commercial manufacturers have found it convenient to use a ring with a build-in PPG-sensor or a finger cuff with a sensor to connect with a smartphone [12,23]. Some studies claim that finger PPG signal is cleaner than signals from wrist PPG [24]. But to our extent of knowledge, there is only a few studies that have assessed the different location of the PPG sensor when estimating HRV [25,26]. One of them assessed the signal quality and found waveforms from finger PPG to have higher quality than waveforms from the wrist. The second study compared HRV features derived from finger and wrist and compared it with HRV features from ECG. They found the performance from wrist and finger PPG to be significantly different in favor of finger PPG [25]. Also, the Shimmer sensors we used are only compatible with measurements on the finger and earlobe. In addition, the fingertip placement is relevant to HRV estimation based on devices for non-invasive pulse wave or blood pressure techniques involving PPG, such as the Finometer® or the successors Nexfin® and Clearsight® monitors.

There were no restrictions on how much the participants were allowed to move during this study. This means that unnecessary disturbance may have been introduced to the PPG measurements. For further research on this topic, the earlobe sensor would be interesting to assess for HRV estimation. The earlobe sensor is supposed to be less prone to motion artifacts and may have lower variability in the skin-sensor interface.

We had the technical issue of signal saturation from the PPG sensor and due to the PPG sensor signal cutoff, it was not possible to assess the PPG-based HRV estimation using the PPG peaks as fiducial points. However, other studies have found the valley or maximum slope points to be more accurate compared to the peak point in HRV estimation [4,27], suggesting that the lack of a peak-based PPG analysis would not have influenced our findings on the performance possibilities of PPG in estimating HRV under different conditions.

It is possible that further improvement of the PPG signal processing and fiducial point detection could improve the HRV estimation, but we believe that our main findings stand pertinent nevertheless: That finger PPG can provide a good estimation of HRV during relaxation and mental stress, that movement artifacts during exercise can cause large errors, and that outlier correction is essential. We are not able to tell to which extent our findings are relevant for PPG sensors in general or the particular sensor used in this study (Shimmer 3 GSR+ unit), as both optics and signal conditioning may vary from device to device.

Measurement of heart rate by PPG during exercise is already challenging due to motion artifacts, and advanced signal processing is needed to provide good estimates [7,28], possibly improved by implementing sensor signals from an accelerometer [29]. Estimates of HRV during exercise would require signal processing for improved fiducial point estimation from disturbed signals caused by motion artifacts, without rejecting or interpolating too many beats as this would reduce the beat-to-beat or high-frequency HRV information. If applicable, it would be easier and more feasible to measure HRV based on a recording period directly after the exercise has ended for studies of HRV using PPG sensors.

## Conclusion

Our results clearly show that finger PPG-based HRV estimation is feasible during relaxation and mild mental stress, but can have large errors even during mild physical exercise. The HRV estimation is very sensitive to outliers in the time-series of interbeat intervals and produces large errors when uncorrected. The maximum slope point of the PPG signal may be the most optimal fiducial point for HRV estimation. SDNN seems to be more robust for PPG-based HRV estimation compared to RMSSD.

## Figures and Tables

**Figure:**
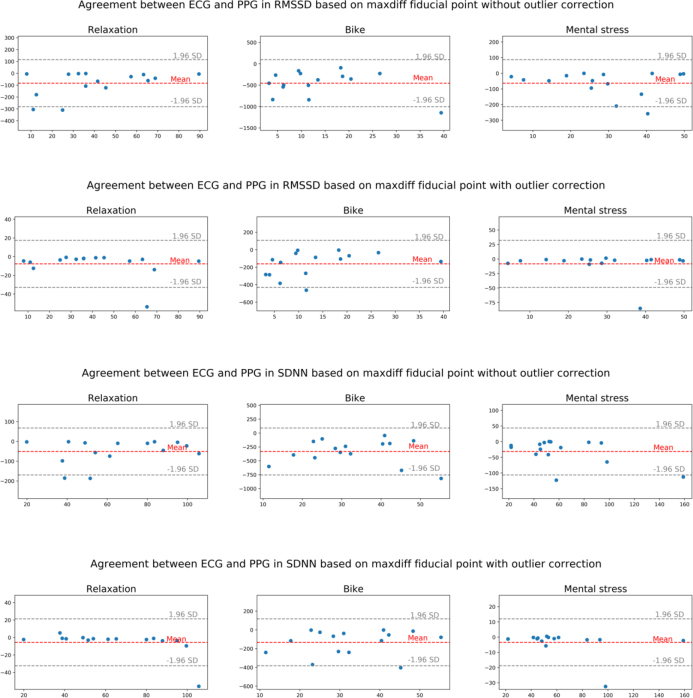


**Figure:**